# Nicotine exacerbates atherosclerosis through a macrophage-mediated endothelial injury pathway

**DOI:** 10.18632/aging.202660

**Published:** 2021-02-24

**Authors:** ChengYu Mao, DongJiu Li, En Zhou, JunFeng Zhang, ChangQian Wang, Chao Xue

**Affiliations:** 1 Department of Cardiology, Ninth People’s Hospital, Shanghai Jiao Tong University School of Medicine, Shanghai 200011, People’s Republic of China

**Keywords:** nicotine, macrophages, reactive oxygen species, endothelial injury, inflammasome

## Abstract

Evidence suggests that nicotine intake promotes atherosclerosis. We enrolled 100 patients with coronary heart disease (CHD) and found that plaque burden, TXNIP expression, and inflammatory chemokine levels were higher in smokers than non-smokers. Additionally, patients with higher TXNIP expression in peripheral blood mononuclear cells (PBMCs) had a higher Gensini Scores and higher plasma IL-1β and IL-18 levels. Treating bone marrow-derived macrophages (BMDMs) with nicotine *in vitro* led to enhanced lipid phagocytosis, chemotaxis, and increased production of reactive oxygen species (ROS), which activated TXNIP/NLRP3 inflammasome signaling and promoted pyroptosis, as evidenced by caspase-1 cleavage and increased production of IL-1β, IL-18, and gasdermin D. Nicotine intake by ApoE^(-/-)^ mice fed a high-fat diet recapitulated those phenotypes. The effects of nicotine on pyroptotic signaling were reversed by N-acetyl-cysteine, a ROS scavenger. Silencing TXNIP *in vivo* reversed the effects of nicotine on macrophage invasion and vascular injury. Nicotine also induced pyroptotic macrophages that contributed to the apoptotic death of endothelial cells. These findings suggest that nicotine accelerates atherosclerosis in part by promoting macrophage pyroptosis and endothelial damage. Therefore, targeting the TXNIP/NLRP3-mediated pyroptotic pathway in macrophages may ameliorate nicotine-induced endothelial damage.

## INTRODUCTION

Smoking is a major risk factor for atherosclerosis and cardiovascular disease [1–4], with even low-tar cigarettes and smokeless tobacco increasing the risk for cardiovascular events [5]. Nicotine is a major component in cigarettes and increasing evidence suggests that it promotes atherosclerosis development [6]. Identifying the mechanisms by which nicotine aggravates atherosclerosis would provide interventions that benefit people exposed to tobacco.

Macrophages are integral cells of the innate immune system and play a crucial role in atherosclerosis progression. At the early stages of the disease, macrophages attempt to clear cholesterol deposits from the intima [7, 8]. However, during the process of atherosclerosis there is a maladaptive, pro-inflammatory response. The macrophages secrete proinflammatory cytokines and chemokines that recruit circulating blood monocytes into the sub-endothelial space, where they phagocytize lipoprotein-derived cholesterol to become foam cells [8, 9].

Inflammasomes composed of NOD-like receptor containing pyrin domain 3 (NLRP3) are highly associated risk factors for deteriorated atherosclerosis [10]. Inflammasomes activate caspase-1, which cleaves the precursors of IL-1β and IL-18. These cytokines are then released from cells through special channels on phospholipid membrane induced by gasdermin D (GSDMD)—a process of pyroptosis [11]. Previous research has shown that ox-LDL and extracellular acidosis exacerbate atherosclerosis by activating the NLRP3 inflammasome [10, 12]. Given this pivotal role of NLRP3 inflammasome in atherosclerosis, it is important to examine whether a connection exists between smoking/nicotine and inflammasome activation.

Smoking could increase the secretion of IL-1 by macrophages [13]. For instance, smokers increased the expression of NLRP3 and IL-1 in peripheral vascular adipose tissue and plasma compared with non-smokers, suggesting that smoking may activate a NLRP3-related pyroptosis pathway [14]. Additionally, nicotine can promote inflammasome activation in endothelial cells [15]. These studies suggest that inflammasome activation plays a key role in nicotine-induced atherosclerosis. However, how nicotine activates inflammasomes in macrophages remains unclear.

Reactive oxygen species (ROS), which is produced by many stimuli, plays a central role in triggering NLRP3 inflammasome formation and activation [16]. Thioredoxin-interacting protein (TXNIP) which belongs to the arrestin superfamily, inhibits the antioxidant activity of the endogenous antioxidant thioredoxin (TRX) by binding. When ROS are produced, they oxidize TRX, resulting in the dissociation of TXNIP. Free TXNIP then binds to NLRP3 to promote inflammasome formation and pyroptosis [17]. Previous studies indicate that smoking increases ROS production [18]. However, evidence of ROS production triggered by nicotine is limited and the role of TXNIP in atherosclerosis is undefined.

We hypothesized that nicotine may trigger inflammasome activation through ROS production, particularly mitochondrial reactive oxygen species (mROS), which is the initiating link of pyroptosis. After ROS activation, TXNIP can dissociate from TRX and bind to NLRP3, further activating NLRP3 inflammasomes. Activated pyroptosis pathways eventually cause GSDMD to have biological effects that aggravate atherosclerosis. In this study, we treated ApoE^(-/-)^ mice and bone marrow-derived macrophages (BMDM) with nicotine and investigated the mechanism of nicotine-triggered BMDM dysfunction.

## RESULTS

### Changes in TXNIP expression and coronary atherosclerosis in smoking versus non-smoking patients

**Figure 1:**
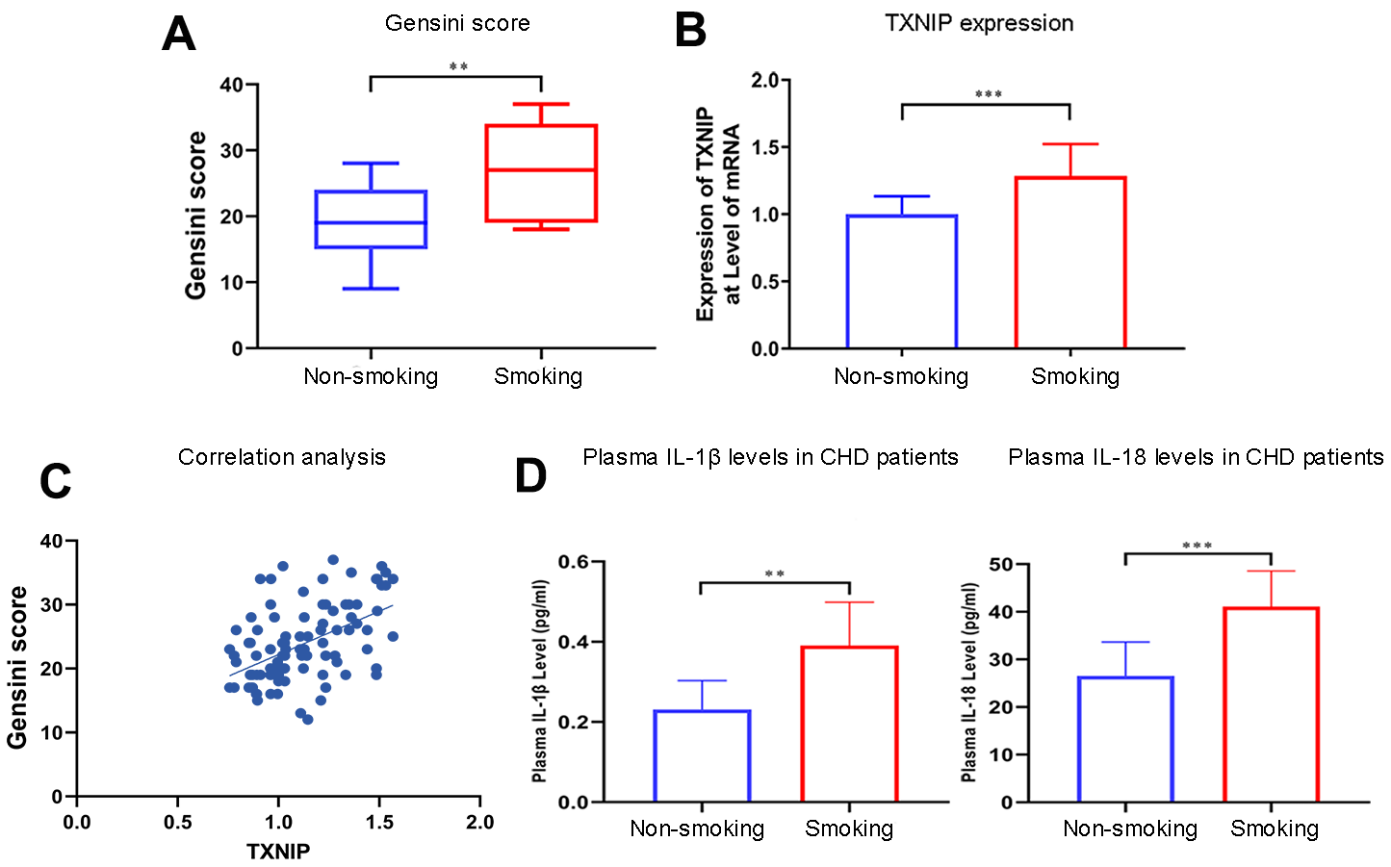
**Smoking aggravates the Gensini score, TXNIP expression, and cytokine secretion in patients with CHD.** (**A**) The Gensini score was evaluated in every patient based on the results of coronary angiography. (**B**) TXNIP mRNA expression was measured in the monocytes of CHD patients. (**C**) Correlation analysis of Gensini score and TXNIP expression. (**D**) IL-1β and IL-18 from plasma in patients with CHD were analyzed. The smoking groups were compared to the non-smoking groups (*P < 0.05, **P < 0.01, ***P < 0.001, ****P < 0.0001).

Patients who smoked had a higher Gensini score (P=0.0028) (Figure 1A) and TXNIP expression at the transcriptional level than non-smoking patients (P=0.0004) (Figure 1B). Additionally, TXNIP expression was positively correlated with the Gensini score of these patients (r=0.5025, Figure 1C). The baseline of patients enrolled in this study is shown in Table 1 (primer sequences are shown in Supplementary Table 1).

**Table 1:** Baseline characteristics and Gensini score of the study population.

**Characteristics**^a^	**All subjects** **(n=100)**	**Smokers** **(n=41)**	**Nonsmokers** **(n=59)**	**P value**
Age (years)	63.5±8.7	63.7±9.6	63.4±8.2	0.866
Male n (%)		37 (90%)	35(59%)	0.001
BMI(kg/m^2^)	25.0±3.2	25.3±3.5	24.8±2.9	0.503
Waist (cm)	85.9±6.0	86.1±6.1	85.9±5.9	0.913
Brinkman index	461±702	916±710	0	0.000
Cigarettes smoked per day	9.5±13.4	23.2±11.0	0	0.000
SBP (mm Hg)	127±15	128±14	125±15	0.343
DBP (mmHg)	82±11	83±10	81±11	0.558
Heart rate (bpm)	73±7	73±8	73±6	0.861
Risk factors n (%)				
Hypertension	66(6%)	28(68%)	38(64%)	0.687
Diabetes mellitus	35(35%)	19(46%)	16(27%)	0.047
Hypercholesterolemia	35(35%)	15(37%)	20(34%)	0.213
Impaired renal function	11(11%)	5(12%)	6(10%)	0.075
Left ventricular ejection fraction (%)	63.7±2.7	63.7±2.5	63.7±2.8	0.875
Total cholesterol (mmol/L)	4.17±0.79	4.20±0.70	4.15±0.86	0.743
Triglycerides (mmol/L)	1.78±0.89	1.92±0.85	1.69±0.93	0.222
HDL-cholesterol (mmol/L)	1.03±0.20	1.05±0.27	1.00±0.14	0.279
LDL-cholesterol (mmol/L)	2.78±0.81	3.00±0.83	2.62±0.77	0.017
Gensini Score	23.47±5.47	27.3±7.07	19.6±5.80	0.0028
TXNIP	1.14±0.20	1.28±0.24	1±0.134	0.0004

Abbreviations: BMI, body mass index; SBP, systole blood pressure; DBP, diastolic blood pressure; HDL, high-density lipoprotein; LDL, low-density lipoprotein; Brinkman index (BI) = (Number of cigarettes smoked per day) x (Number of years smoked).

^a^Values are mean (standard deviation) for continuous variables and percentages for categorical variables unless otherwise specified.

### Nicotine enhances ROS production in BMDMs independent of TXNIP

**Figure 2:**
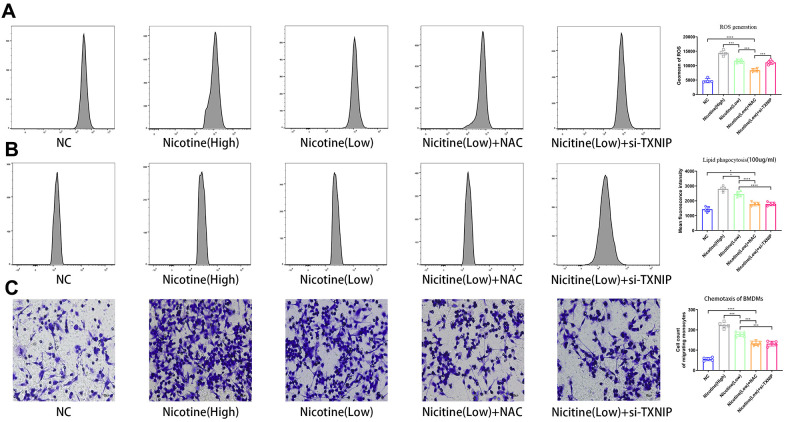
**Nicotine exacerbates BMDM dysfunction by ROS production, lipid phagocytosis, and chemotaxis.** (**A**) ROS production was measured in BMDMs by the ROS probe Relative (APC channel detected by flow cytometry), which increased with the nicotine concentrations (*P < 0.05, **P < 0.01, ***P < 0.001, ****P < 0.0001, n=4). (**B**) Lipid phagocytosis was measured in BMDMs by flow cytometry in the PE channel, which increased with nicotine concentration (*P < 0.05, **P < 0.01, ***P < 0.001, ****P < 0.0001, n=4). (**C**) chemotaxis towards medium containing CCL2 (50 ng/mL) was significantly increased in BMDMs pretreated with nicotine, as determined by a transwell assay (*P < 0.05, **P < 0.01, ***P < 0.001, ****P < 0.0001, n=5).

ROS production in BMDMs was measured using a ROS probe (ab113851, Abcam, USA) and flow cytometry in the APC channel according to the manufacturer’s instructions (Figure 2A). ROS production increased in BMDMs following an increase in nicotine concentrations [nicotine (high) vs. nicotine (low), P<0.0001; nicotine (low) vs. NC, P=0.001]. However, knocked-down TXNIP had no effect on ROS generation [nicotine (low) + si-TXNIP vs. nicotine (low), P=0.8796].

### Nicotine promotes lipid phagocytosis of BMDMs that is mediated by ROS-TXNIP

BMDMs were incubated with Dil-ox-LDL (100 μg/mL) for 6 h. The cells were then washed and harvested. The ox-LDLs phagocytized in BMDMs were measured by flow cytometry using the PE channel (Figure 2B). Lipid phagocytosis increased in BMDMs with increasing nicotine concentrations [nicotine (high) vs. nicotine (low), P=0.0168; nicotine (low) vs. NC, P<0.0001].

When NAC was used to neutralized ROS and block its function, the fluorescence intensity of lipid in BMDMs decreased [nicotine (low) vs. nicotine (low) + NAC, P<0.0001]. When TXNIP expression was suppressed by si-TXNIP in BMDMs, lipid phagocytosis of BMDMs was also inhibited [nicotine (low) + si-TXNIP compared to the nicotine (low) group + NAC, P=0.9987].

### The role of nicotine in macrophage migration

To investigate the effect of nicotine on macrophage migration, we performed a macrophage chemotaxis assay on BMDMs (Figure 2C). The transwell assay revealed that the migration capacity of BMDMs increased with nicotine concentration [nicotine (high) vs. nicotine (low), P=0.0006; nicotine (low) vs. NC, P<0.0001]. Likewise, the migration capacity of BMDMs was suppressed due to NAC administration to eliminate its ROS production [nicotine (low) vs. nicotine (low) + NAC, P=0.0002; nicotine (low) vs. nicotine (low) + si-TXNIP, P=0.0002;].

### BMDMs pre-treated with nicotine aggravate endothelial cell apoptosis

**Figure 3:**
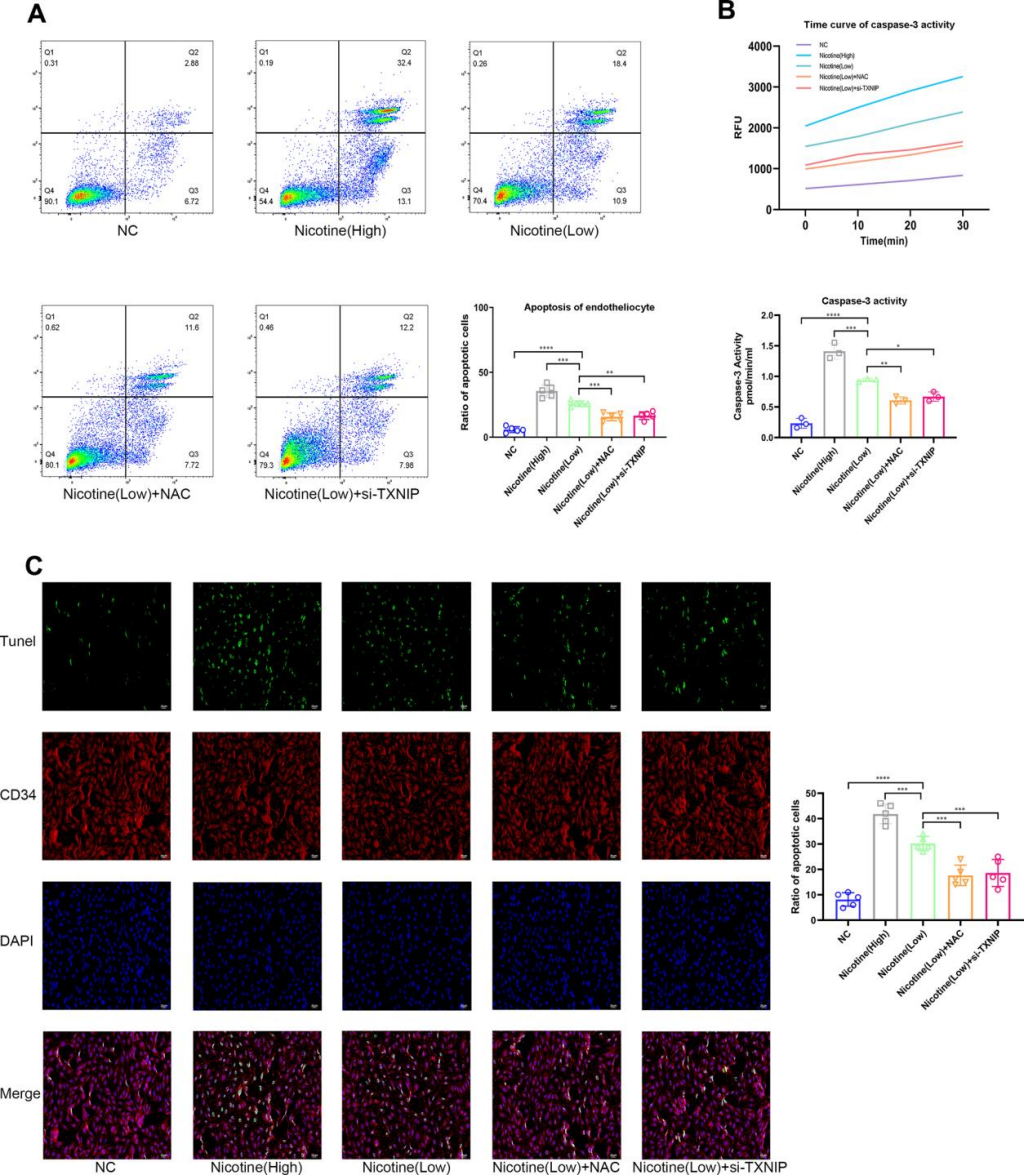
**Nicotine exacerbates the pro-apoptotic effect of BMDMs on endotheliocytes.** (**A**) Proportion of apoptotic endotheliocytes co-incubated with BMDMs pretreated with nicotine were analyzed by an AnnexinV-PI assay (*P < 0.05, **P < 0.01, ***P < 0.001, ****P < 0.0001, n=5). (**B**) A caspase-3 activity assay was employed to detect the apoptotic degree of endotheliocytes co-incubated with BMDMs pretreated with nicotine (*P < 0.05, **P < 0.01, ***P < 0.001, ****P < 0.0001, n=3). (**C**) The apoptotic degree of endotheliocytes induced by BMDMs pretreated with nicotine was analyzed by a TUNEL assay and binding of the CD34 antibody to endotheliocytes (*P < 0.05, **P < 0.01, ***P < 0.001, ****P < 0.0001, n=5).

Mice aortic endothelial cells were co-cultured with BMDMs that were pretreated with different concentrations of nicotine for 24 h (the supernatant containing nicotine was removed). Then, mice aortic endothelial cells were washed and harvested for Annexin V/PI (APC and PI channel), TUNEL, and Caspase-3 activity to detect the apoptotic level (Figure 3A–3C). As the pretreated dose of nicotine increased, the pro-apoptotic effect of BMDMs on mice aortic endothelial cells was elevated [Annexin V/PI: nicotine (high) vs. nicotine (low), P=0.0002; nicotine (low) vs. NC, P<0.0001; TUNEL: nicotine (high) vs. nicotine (low), P=0.0002; nicotine (low) vs. NC, P<0.0001; Caspase-3 activity: nicotine (high) group vs. nicotine (low), P=0.0002; nicotine (low) vs. NC, P<0.0001].

Moreover, ROS and TXNIP were inhibited by NAC and si-TXNIP in BMDMs, respectively, while the pro-apoptotic effect of BMDMs on mice aortic endothelial cells induced by nicotine was ameliorated [Annexin V/PI: nicotine (low) vs. nicotine (low) + NAC, P=0.0005; nicotine (low) vs. nicotine (low) + si-TXNIP, P=0.0012; TUNEL: nicotine (low) vs. nicotine (low) + NAC, P=0.0004; nicotine (low) vs. nicotine (low) + si-TXNIP, P=0.001; Caspase-3 activity: nicotine (low) vs. nicotine (low) + NAC, P=0.0037; nicotine (low) vs. nicotine (low) + si-TXNIP, P=0.0148].

These results indicate that nicotine-treated BMDMs aggravated the apoptosis of mice aortic endothelial cells in a paracrine manner by releasing cytokines that were mediated through the ROS-TXNIP pathway.

### The mechanism for nicotine’s effect on BMDMs pyroptosis activation

**Figure 4:**
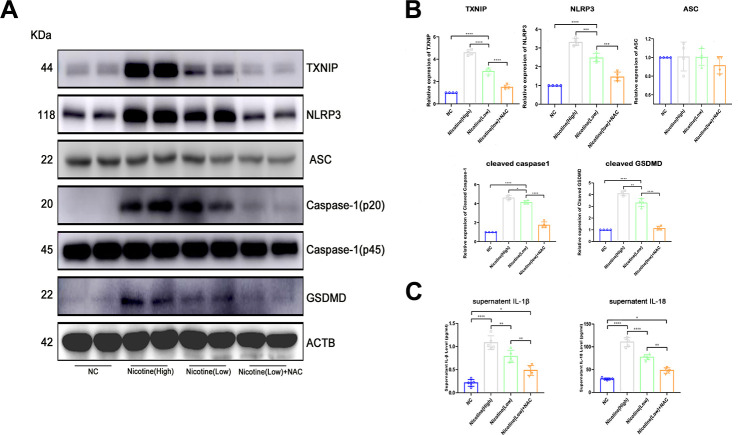
**Nicotine activates the pyroptosis pathway in BMDMs by upregulating ROS production.** (**A**, **B**) Western blot was used to detect the expression of TXNIP, NLRP3, ASC, caspase-1, and GSDMD. The average ratios were calculated based on gray intensity analysis (*P < 0.05, **P < 0.01, *** P < 0.001, **** P < 0.0001, n = 4). (**C**) IL-1β and IL18 in the supernatant of BMDMs treated with or without nicotine were measured by ELISA (*P < 0.05, **P < 0.01, ***P < 0.001, ****P < 0.0001, n=5).

Based on the finding of nicotine stimulating ROS production and TXNIP upregulation, we speculated that activation of the pyroptosis pathway played a critical role in nicotine-induced monocyte/macrophage dysfunction (Figure 4A, 4B). To clarify the downstream ROS and TXNIP, we assayed pyroptosis-associated protein expression, including NLRP3, ASC, caspase-1, and gasdermin D(GSDMD). TXNIP expression significantly increased in BMDMs treated with increasing doses of nicotine [nicotine (high) vs. nicotine (low), P<0.0001; nicotine (low) vs. NC, P<0.0001].

As for NLRP3 and caspase-1, which is a core component of inflammasomes, expression significantly increased [NLRP3: nicotine (high) vs. nicotine (low), P=0.0001; nicotine (low) vs. NC, P<0.0001; caspase-1: nicotine (high) vs. nicotine (low), P=0.0386; nicotine (low) vs. NC group, P<0.0001].

Moreover, GSDMD, an executive molecule of pyroptosis that is cleaved by caspase-1, was upregulated due to NLRP3 inflammasome formation triggered by nicotine [nicotine (high) vs. nicotine (low), P=0.0079; nicotine (low) vs. NC, P<0.0001].

However, when ROS of BMDMs was neutralized by NAC, NLRP3 inflammasome formation and pyroptosis activation were inhibited [TXNIP: nicotine (low) vs. nicotine (low) + NAC group, P<0.0001; NLRP3: nicotine (low) vs. nicotine (low) + NAC, P<0.0001; caspase-1: nicotine (low) vs. nicotine (low) + NAC, P=0.0001; GSDMD: nicotine (low) vs. nicotine (low) + NAC, P<0.0001].

Taken together, these results indicate that the mechanism of nicotine-induced monocyte/macrophage dysfunction was at least partially due to NLRP3 inflammasome formation and pyroptosis activation.

### ELISA for IL-1β and IL-18

Concentrations of IL-1β and IL-18 were detected in the supernatant of BMDMs treated with nicotine, plasma of ApoE^(-/-)^ mice administrated with nicotine, and CHD patients. IL-1β and IL-18 concentrations in the plasma of smoking CHD patients were much higher than that of CHD patients who were never exposed to smoking (IL-1β: non-smoking vs. smoking, P=0.0011; IL-18: non-smoking vs. smoking, P=0.0003; Figure 1D).

IL-1β and IL-18 in the supernatant of BMDMs treated with nicotine were much higher than that of the NC group (Figure 4C). These effects could be reversed by ROS scavenger N-acetyl-cysteine (NAC), as the expression of IL-1β and IL-18 were down-regulated [IL-1β: HFD + nicotine (high) vs. HFD + nicotine (low), P=0.0055; HFD + nicotine (low) vs. HFD + nicotine (low) + NAC, P=0.0046; IL-18: HFD + nicotine (high) group vs. HFD + nicotine (low), P<0.0001; HFD + nicotine (low) vs. HFD + nicotine (low) + NAC, P<0.0001].

**Figure 5:**
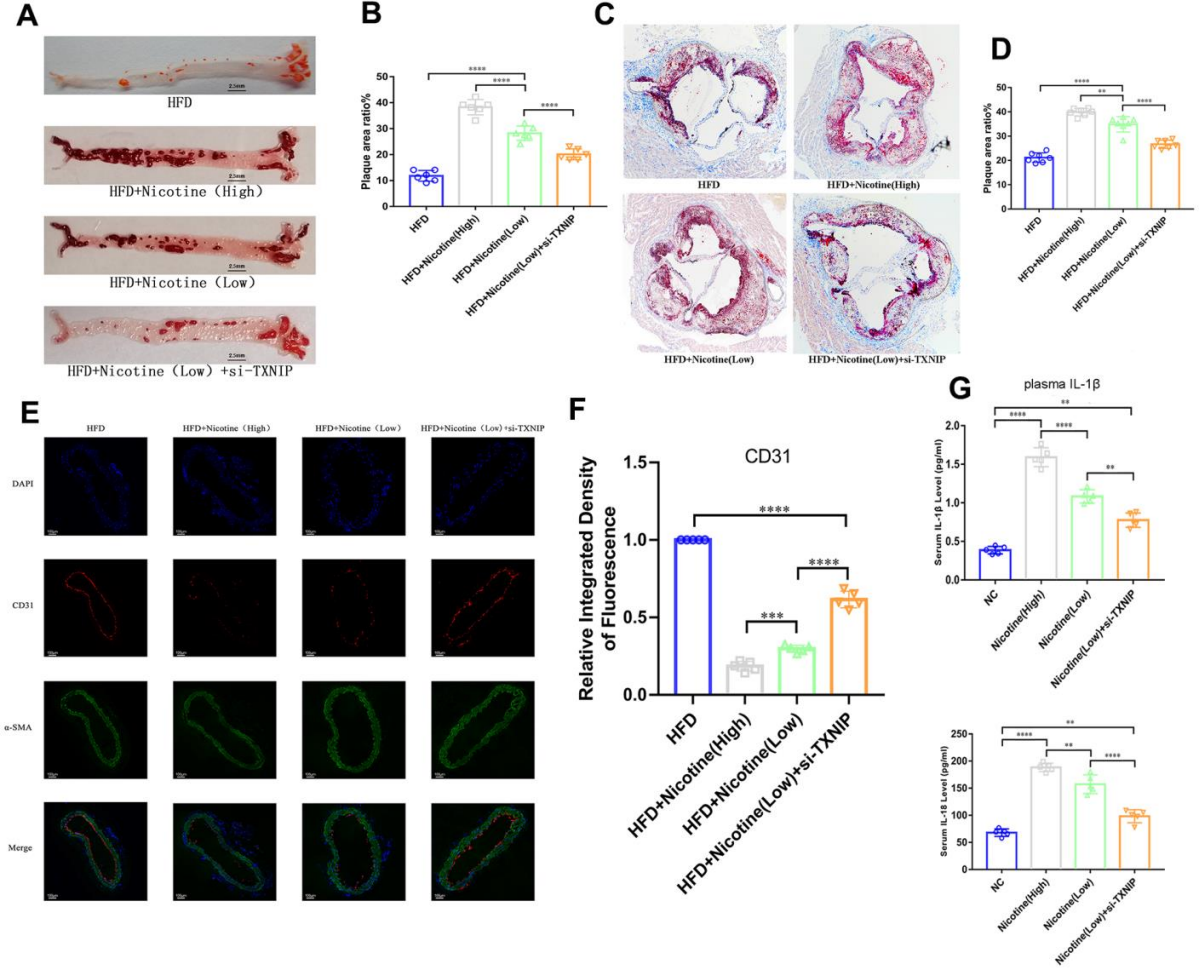
**Nicotine exacerbates endothelial injury and atherosclerosis.** (**A**, **B**) ApoE^(-/-)^ mice fed a HFD and different concentrations of nicotine for 12 weeks, atherosclerotic lesion areas in the longitudinal-section of the aorta were estimated by Oil Red O staining (*P < 0.05, **P < 0.01, ***P < 0.001, ****P < 0.0001, n=6). (**C**, **D**) Atherosclerotic lesion areas in the cross-sections of the aortic root were estimated by Oil Red O staining (*P < 0.05, **P < 0.01, ***P < 0.001, ****P < 0.0001, n=6). (**E**, **F**) Vascular endothelium integrity of the brachiocephalic trunk cross-section was estimated through CD31 fluorescence intensity, with the degree of endothelial injury indicated by a lower fluorescence intensity (*P < 0.05, **P < 0.01, ***P < 0.001, ****P < 0.0001, n=5). (**G**) IL-1β and IL-18 from the plasma of ApoE^(-/-)^ mice fed a HFD and different concentrations of nicotine for 12 weeks were analyzed (*P < 0.05, **P < 0.01, ***P < 0.001, ****P < 0.0001, n=5).

Concentrations of IL-1β and IL-18 in the plasma of ApoE^(-/-)^ mice administrated with nicotine were much higher than that of the NC group (Figure 5G). In addition, silencing TXNIP by AAV harboring si-TXNIP in ApoE^(-/-)^ mice could reduce IL-1β and IL-18 in the plasma of ApoE^(-/-)^ mice [IL-1β: HFD + nicotine (high) vs. HFD + nicotine (low), P<0.0001; HFD + nicotine (low) vs. HFD + nicotine (low) + si-TXNIP, P=0.0068; IL-18: HFD + nicotine (high) vs. HFD + nicotine (low), P=0.0089; HFD + nicotine (low) vs. HFD + nicotine (low) + si-TXNIP, P<0.0001].

### Nicotine exacerbates atherosclerotic lesions in ApoE^(-/-)^ mice, which is mediated by TXNIP

ApoE^(-/-)^ mice were divided into four groups: HFD, HFD + nicotine (high), HFD + nicotine (low), and HFD+nicotine (low) + si-TXNIP (AAV harboring si-TXNIP). All ApoE^(-/-)^ mice were administrated with the experimental processing mentioned above for 12 weeks. We performed Oil Red O staining in the aortic root cross-sections and longitudinal-sectioning of the aortas. The HFD-induced atherosclerosis model was established based on ApoE^(-/-)^ mice fed the HFD diet for 12 weeks having a larger atherosclerotic plaque area than that of ApoE^(-/-)^ mice fed a normal diet (Supplementary Figure 1A, 1B, P=0.004). The results demonstrated that 12 weeks of nicotine-loading exacerbated atherosclerotic plaque. As the intake of nicotine increased, the proportion of atherosclerotic plaque area was also increased in the longitudinal and cross sections. As shown in Figure 5A, 5B, the plaque area in the longitudinal aorta section in the HFD + nicotine (low) group was larger than that of the HFD group (P<0.0001). Likewise, the HFD + nicotine (high) group expressed more severe plaque conditions than that of the HFD + nicotine (low) group (P<0.0001). After inhibiting TXNIP expression in mice, the HFD + nicotine (low) + si-TXNIP group showed a trend of improved atherosclerotic plaque lesions compared to the HFD + nicotine (low) group (P<0.0001).

A similar pattern of plaque lesions (Figure 5C, 5D) was seen in the aortic root cross sections. The proportion of plaque/cross-sectional lumen area in the HFD + nicotine (low) group was higher than that of the HFD group (P<0.0001). The HFD + nicotine (high) group suffered more significant plaque burden than the HFD + nicotine (low) group (P=0.0015). After silencing TXNIP, the plaque burden of the HFD + nicotine (low) + si-TXNIP group was improved compared to the HFD + nicotine (low) group (P<0.0001).

### Nicotine aggravates endothelium injury in ApoE^(-/-)^ mice with a HFD diet

The integrity of the vascular endothelium in the brachiocephalic trunk cross-section was estimated through labeling CD31 (endothelial cells) and α-SMA positive cells (vascular smooth muscle). The fluorescence intensity of CD31 in the cross-section was suppressed as the concentration of nicotine increased (Figure 5E, 5F). However, once TXNIP expression was inhibited, the fluorescence intensity of CD31 increased [HFD + nicotine (high) vs. HFD + nicotine (low), P=0.0006; HFD + nicotine (low) vs. HFD + nicotine (low) + si-TXNIP, P<0.0001]. The continuity of CD31 fluorescence, which represents the degree of vascular endothelial coverage in vessel lumen, was deteriorated with increasing concentrations of nicotine. Silencing TXNIP could block this effect of nicotine injury on the vascular endothelium.

### Nicotine enhances macrophage chemotaxis and TXNIP expression in plaque

**Figure 6:**
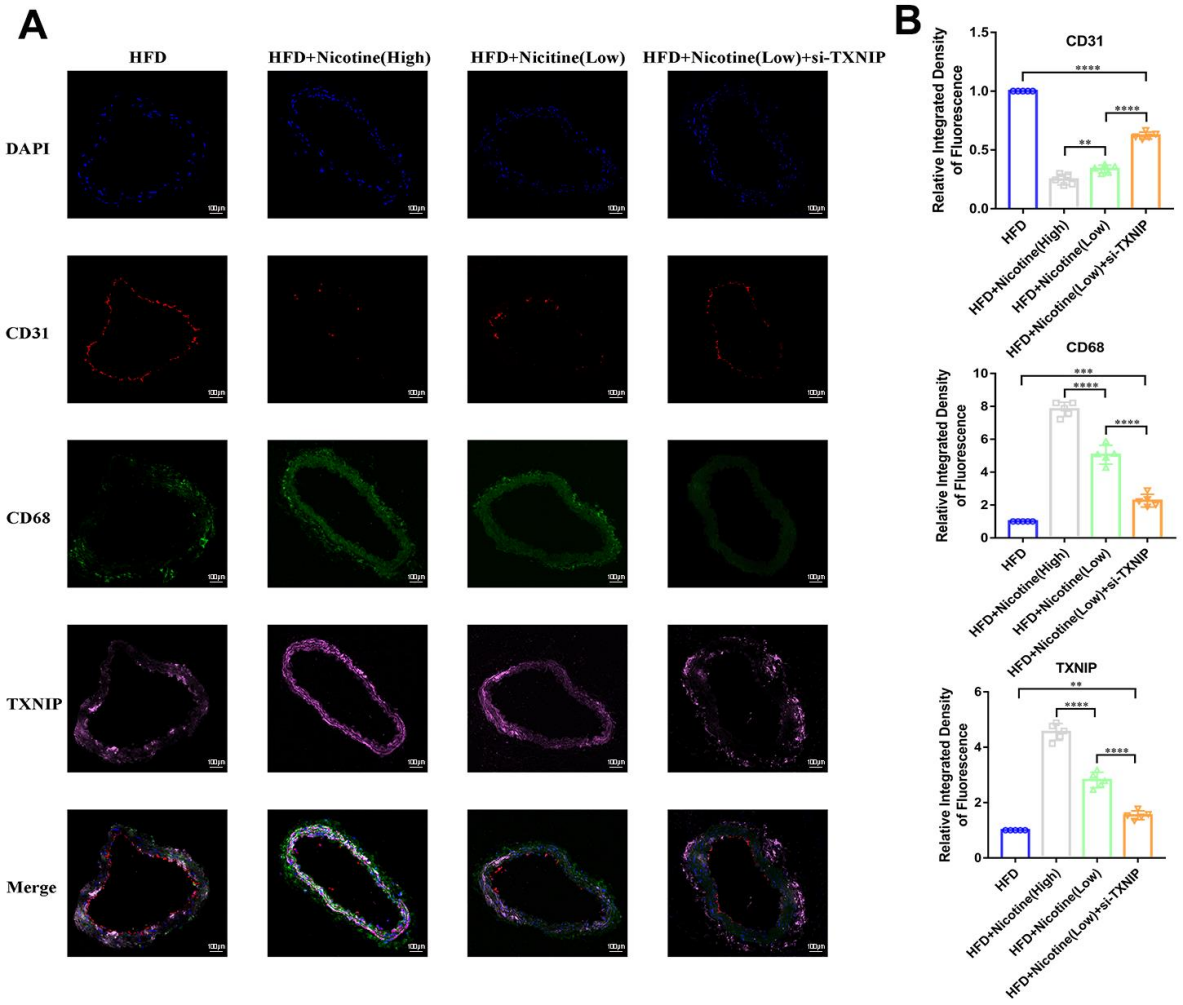
**Nicotine enhances macrophage chemotaxis and TXNIP expression in plaque.** (**A**, **B**) Triple immunofluorescence for CD68, TXNIP, and CD31 was used to analyze macrophage chemotaxis, TXNIP expression in vascular wall tissues, and vascular endothelium integrity, respectively, and their connection in the plaque of brachiocephalic trunk cross-sections (*P < 0.05, **P < 0.01, ***P < 0.001, ****P < 0.0001, n=5).

Figure 6A, 6B demonstrates that nicotine promoted CD68^+^ expression in the brachiocephalic trunk cross-section vessel wall, which indicates that CD68^+^ cells (mainly macrophages) had stronger chemotaxis to the lipids deposited in the vessel wall under HFD conditions. The integrated density of CD68 fluorescence increased with an increase in nicotine concentration [HFD + nicotine (high) vs. HFD + nicotine (low), P<0.0001; HFD + nicotine (low) vs. HFD, P<0.0001]. Silencing TXNIP inhibited CD68 expression in the brachiocephalic trunk [HFD + nicotine (low) vs. HFD + nicotine (low) + si-TXNIP, P<0.0001]. These findings suggest that nicotine promoted chemotaxis of macrophages partly through regulating TXNIP expression. In addition, TXNIP expression in the vessel wall was elevated with increasing nicotine dose [HFD + nicotine (low) vs. HFD, P<0.0001; HFD + nicotine (high) vs. HFD + nicotine (low), P<0.0001]. Similar to the results of Figure 4E, the integrity and fluorescence intensity of CD31 (endothelial cells) was suppressed with increased concentrations of nicotine. This trend was inhibited by silencing TXNIP expression.

## DISCUSSION

Nicotine directly promotes endothelial damage and dysfunction, thus driving atherosclerotic plaque formation [15, 19, 20]. Our study adds insight into the complex pathophysiology of atherosclerosis by demonstrating nicotine-induced pyroptotic macrophages promote endothelial cell apoptosis.

Atherosclerotic cardiovascular diseases are the leading cause of death worldwide. Various risk factors have been identified for atherosclerosis. Among which, cigarette smoking is a major public health concern. Nicotine is an alkaloid found primarily in tobacco and is mostly absorbed from smoking [21]. Numerous studies demonstrated that nicotine intake promotes atherosclerosis development and progression [6, 15, 22, 23]. Consistent with these studies, our data showed that nicotine intake significantly increased lipid deposition and plaque area as revealed by Oil Red O staining.

Accumulating evidence suggests that risk factors for atherosclerosis could activate pyroptosis in macrophages and endothelial cells. Additionally, NLRP3 inflammasome-mediated pyroptosis is positively correlated with plaque instability and vascular inflammation [24]. However, whether nicotine contributes to atherosclerotic plaque formation by mediating macrophage pyroptosis remains largely unknown. A previous study showed that cholesterol crystals were the initiating factor that triggered NLRP3 inflammasome activation in phagocytes [25]. Since then, initiating factors that activate NLRP3 inflammasome in macrophages have been identified, including oxidized low-density lipoprotein (ox-LDL), triglyceride, and homocysteine (Hcy) [26–28]. Other studies also showed that vascular endothelial cells could undergo NLRP3 inflammasome-mediated pyroptosis induced by nicotine, calcium, palmitic acid, ox-LDL, as well as Hcy [15, 29–31]. We found that nicotine-treated macrophages had increased ROS production and activated TXNIP/NLRP3/caspase-1 signaling. The macrophages also showed enhanced adherence and invasiveness that promoted endothelial cell apoptosis, which in turn could accelerate atherosclerotic plaque formation. As far as we know, this is the first study to show that nicotine indirectly promotes endothelial damage and dysfunction.

ROS generation activates NLRP3 inflammasome signaling [32, 33]. TXNIP was identified as a binding partner of reduced thioredoxin (TRX), which interacts with TXNIP through its redox-active domain [34, 35]. High concentrations of ROS promoted the dissociation of TXNIP from TRX, and also prompted dissociated TXNIP to interact with NLRP3, which activated downstream NLRP3 inflammasome signaling and the release of pro-inflammatory cytokines such as IL-1β [35]. Some studies showed that overexpressed TXNIP could lead cellular apoptosis as well as participate in cardiotoxicity and degeneration [36, 37]. Other studies indicated that TXNIP is involved in TLR4/NF-κB/NLRP3 inflammasome signaling in cardiomyocytes and cardiac endothelial cells [38, 39]. Our study showed that nicotine intake increased ROS production, thereby inducing oxidative stress and activating TXNIP/NLRP3 inflammasome signaling in macrophages. Neutralization of ROS by NAC or TXNIP knockdown diminished inflammasome activation and pro-inflammatory cytokines production, ameliorating the pyroptotic and apoptotic death of macrophages and endothelial cells.

**Figure 7:**
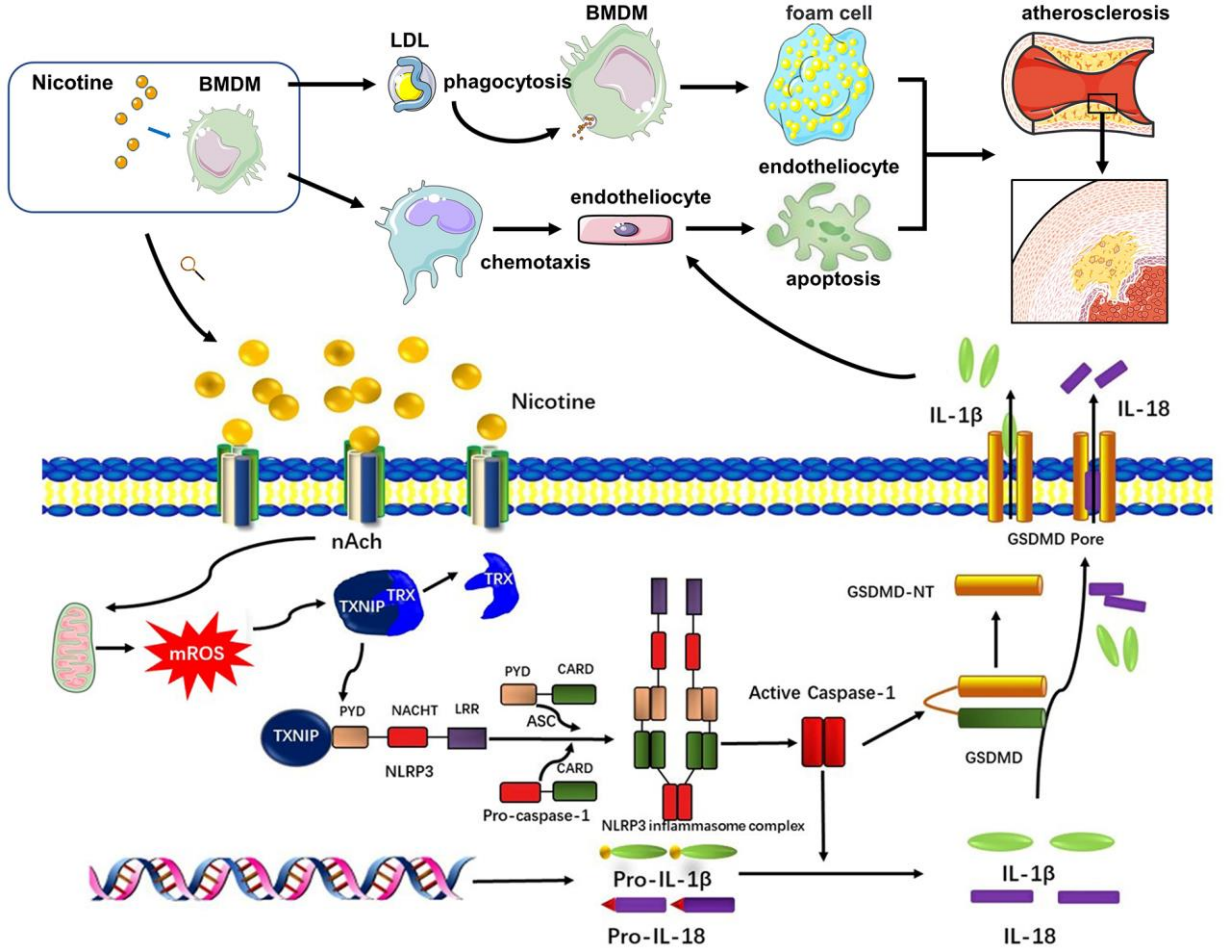
**Signaling pathway diagram.** Nicotine elevated mROS production through binding to nAch receptors on the surface of macrophages. mROS upregulated the expression of TXNIP, which was binded to and inhibited activation of TRX leading to formation of NLRP3 inflammasome. NLRP3 inflammasome was the key to open the process of pyroptosis, which eventually released cytokines, mainly IL-1β, IL-18, caused by GSDMD. Under the influence of nicotine, dysfunction of macrophages mainly manifested in two aspects. One was that nicotine increased LDL-phagocytosis of macrophages, which accelerated the formation of foam cells. The second one was that nicotine activated pyroptosis process through evaluating ROS production to release more cytokines which inducing endothelial injury.

Endothelial cells cover the intima of the blood vessels and are continuously exposed to danger signals related to atherosclerosis in the circulation. Endothelial dysfunction is an initial event responsible for monocyte recruitment in atherosclerosis [40]. Elevated nicotine levels in the blood stream could directly promote endothelial cell damage, as shown in a previous study [15]. However, whether damaged endothelial cells are caused by other dysfunctional cell types or proinflammatory cytokines and chemokines, remains unexplored. A previous study indicated that supernatants from nicotine-treated mast cells promoted macrophage foam cell formation as well as increased production of a variety of pro-inflammatory cytokines from activated macrophages. Our study adds further insight into the complex cell-cell interactions during atherogenesis by showing nicotine-induced pyroptotic macrophages contributes to the apoptotic death of endothelial cells, which could further aggravate atherosclerosis progression (Signaling pathway diagram in the study was concluded in Figure 7).

In summary, our study showed for the first time that the pro-atherosclerotic property of nicotine is its ability to increase ROS production, activate TXNIP/NLRP3 inflammasome signaling, and cause pyroptosis in macrophages. The pyroptotic macrophages exhibited enhanced adherence and invasiveness and further contributed to endothelial cells apoptosis. Thus, targeting the TXNIP/NLRP3-mediated pyroptotic pathway in macrophages may ameliorate nicotine-induced endothelial damage.

## MATERIALS AND METHODS

### Isolation of human primary peripheral blood monocytes

Peripheral human blood was obtained from patients undergoing coronary angiography at the Department of Cardiology, Shanghai Ninth People's Hospital. Patients were divided into the smoking (mean smoking index which is also the Brinkman index ≥ 200) and non-smoking groups according to their detailed medical history. The Gensini score was evaluated for all patients based on the coronary angiography results. Mononuclear cells were isolated for TXNIP expression analysis at the transcriptional level. The clinical study was approved by the hospital ethics review board of Shanghai Ninth People’s Hospital, Shanghai Jiao Tong University School of Medicine.

### Animal studies and mouse model of atherosclerosis

All animal procedures after appraisals and feasibility studies were approved by the Shanghai Ninth People’s Hospital institutional ethics committee (SH9H-2018-A45-2). All experimental contents acted in accordance with the guidelines of the Directive 2010/63/EU of European Parliament. Six-week-old male specific pathogen free (SPF), wild type (WT), and apolipoprotein E knockout (ApoE^(-/-)^) C57BL/6 mice were purchased from the Experimental Animal Center of Shanghai Ninth People’s Hospital. The mice were fed normally under SPF conditions in the experimental animal center until they were 8 weeks old.

A total of 80 healthy (WT C57BL/6) male mice and 40 ApoE^(-/-)^ C57BL/6 male mice (8 weeks old, ~24 g) were kept under SPF experimental animal feeding surroundings (temperature, 20-24° C; humidity, 50-60). Peripheral blood mononuclear cells (PBMC) and bone marrow mesenchymal stem cells were extracted from WT mice. ApoE^(-/-)^ mice were fed a high fat diet (HFD; 16.9% fat, 1.3% cholesterol, 21.1% crude protein, and 46.5% carbohydrates) for 12 weeks to construct a mouse model of atherosclerosis. Simultaneously, mice (8 weeks old) were administrated with water containing 100 μg/mL (high concentration) and 30 μg/mL (low concentration) of nicotine for 12 weeks [6]. Once the model of atherosclerosis was established (20 weeks old), mice were euthanized by excessive carbon dioxide inhalation. Aorta from the ascending aorta to the arteria iliaca communis and hearts were collected for Oil Red O staining and immunofluorescence. ApoE^(-/-)^ mice were randomly divided into four groups: HFD (HFD diet, n=8), HFD + Nicotine (high) (HFD diet and water with 100 μg/mL nicotine, n=8), HFD + Nicotine (low) (HFD diet and water with 30 μg/mL nicotine, n=8), HFD + Nicotine (low)+si-TXNIP (HFD diet and water with 30 μg/mL nicotine + si-TXNIP, n=8, the si-TXNIP sequence is shown in section 2.12).

### Cell culture

Bone marrow-derived macrophages (BMDMs) were isolated from the bone marrow of 4 to 6-week-old WT mice as previously described [41]. Briefly, bone marrow cells taken from tibiae and femurs were incubated in DMEM/F12 medium containing 10% (v/v) FBS, 1% penicillin/streptomycin, and 50 ng/mL of M-CSF. Non-adherent cells were removed, and adherent cells were incubated for an additional three days. After reaching confluence, the BMDMs were harvested and confirmed with F4/80 and CD11b expression by flow cytometry. For the *in vitro* study, BMDMs were divided into five groups: NC group (BMDMs without any treatment); nicotine (high) group (BMDMs pretreated with 3 μM nicotine); nicotine (low) group (BMDMs pretreated with 1 μM nicotine); nicotine (low) + NAC group (BMDMs pretreated with 1 μM nicotine and 5 mM NAC); nicotine (low) + si-TXNIP group (BMDMs pretreated with 1 μM nicotine and 100 nM si-TXNIP).

### Macrophage lipid phagocytosis

BMDM cells were treated with Dil-ox-LDL (100 μg/mL) and incubated for 6 h according to the protocols provided by manufacturers. BMDMs were harvested and the red fluorescence intensity of Dil-ox-LDL (excitation 549 nm, emission 565 nm) endocytosed in the cell was estimated through flow cytometry (CytoFLEX S, Beckman, USA).

### Reactive oxygen species (ROS) measurement

ROS production in BMDMs was measured using a ROS Detection Assay Kit (ab113851, Abcam, USA) and flow cytometry according to the manufacturer’s instructions. The data were analyzed with Flowjo 10 software. BMDM cells were seeded at 1×10^6^ cells/well into sterile 12-well plates incubated with different concentrations of nicotine with or without NAC and kept for 24 h at 37° C in a 5% CO_2_ incubator. BMDMs were detached and collected in tubes. After BMDMs were stained with DCFDA (excitation 485 nm, emission 535 nm) for 30 min (without washing), ROS in the cells were analyzed by a flow cytometer (CytoFLEX S, Beckman, USA).

### Quantification of atherosclerosis

At the time of sacrifice, the mice were weighed and their plasma collected for further analysis. Atherosclerotic area was quantified by staining the aortic plaque with Oil Red O as previously described [42]. Atherosclerotic lesion sizes were assessed using commercial software (Image Pro Plus 6.0, Cybernetics, USA) and expressed as the percentage of plaque area relative to the total intimal area. The aortic root was also sectioned for Oil Red O staining. Plaque lesion size was assessed in the same manner and expressed as the percentage of plaque area relative to the cross-sectional luminal area.

### Immunostaining examination

The harvested aortic roots were embedded and sliced. Quantitative immunostaining was performed for macrophages, vascular endothelial cells, and smooth muscle cells in frozen slides of the aorta using an anti-CD68 antibody (ab53444, Abcam, USA), anti-α-SMA antibody (ab7817, Abcam, USA), anti-TXNIP antibody (ab210826, Abcam, USA), and anti-CD31 antibody (ab124432, Abcam, USA). Macrophage, smooth muscle cell, vascular endothelial cell, and TXNIP staining was expressed as the fluorescence intensity of the CD68-, α-SMA-, CD31-, and TXNIP-positive areas respectively.

### Enzyme-linked immunosorbent assay (ELISA)

BMDMs were pretreated with different concentrations of nicotine (1μM, 3 μM) with or without NAC (5 mM) for 24 h and the cell supernatants were collected for further analysis. IL-1β and IL-18 levels were assayed in the cell supernatants and plasma of CHD patients using commercially available ELISA kits (R&D Systems, Minneapolis, USA) according to the manufacturer’s instructions.

### Macrophage migration assay

The chemotaxis of BMDMs was assessed *in vitro* using a transwell chamber migration assay. BMDMs (1×10^5^ cells) were plated onto a transwell upper chamber (8-μm pore size, Millipore) and allowed to migrate across the porous filter for 4 h at 37° C towards CCL2 (50 ng/mL, PeproTech, Inc.). Migrated BMDMs on the bottom of the filter were stained with crystal violet and counted using a fluorescence microscope. The number of cells was determined in five random fields (magnification × 200) for each experiment.

### Endothelial cells apoptosis assay

Mice aortic endothelial cells was isolated from 8 week-old wild type C57BL/6 mice as previously described [43]. Briefly, mice aorta was cut into 1 mm rings. Each segment was seeded onto a growth factor-reduced matrix with the endothelium facing down on the plate. Every piece of endothelium was cultured in endothelial cell growth medium for 3-4 days. The endothelial cells started extending on day 2. These segments of aorta were then removed, and the cells cultured until they reached confluence. Endothelial cells were used for experiments after two passages. Endothelial cells were co-cultured with BMDM in a 0.4 μm 12 well-transwell system. BMDMs were pretreated with different concentrations of nicotine (1 μM, 3 μM) with or without NAC (5 mM) for 24 h, then they were harvested and seeded onto the upper compartment of the transwell system. Endothelial cells were seeded onto the lower compartment of the transwell system and co-cultured with BMDM for 24 h. After the endothelial cells were harvested, their apoptosis levels were evaluated by Annexin V-PI (AD10, Dojindo, Japan), TUNEL (11684795910, Roche, USA, and caspase-3 activity (ab252897, Abcam, USA). Experimental procedures followed the manufacturer's instructions.

### RNA isolation and analysis

Total RNA from human peripheral blood monocytes was extracted with RNAiso Plus extraction reagent following the manufacturer's instructions (No. 9108, Takara, Dalian, China). Total RNA was quantified on a NanoDrop ND-2000 (Thermo Fisher Scientific, USA). For mRNA expression analysis, cDNA was synthesized with reverse transcriptase (TaKaRa Biotechnology, Otsu, Japan) and real-time PCR was performed with TB Green® Premix Ex Taq™ II (RR820L, Takara, Dalian, China) through an ABI-7500 Real-Time PCR Detection System (Applied Biosystems, USA). GAPDH was used as an internal control. The primer sequences used for qPCR are listed in the Supplementary Material. The relative gene expression level was calculated with the comparative 2 ^-∆∆CT^ method and normalized to GAPDH expression.

### *In vivo* and *in vitro* RNA interference

AAV9 expressing green fluorescent protein (GFP) alone, or harboring siRNA targeting TXNIP (si-TXNIP) or a negative control (si-NC), were synthesized and produced by JiKai (Shanghai, China) according to the manufacturer’s instructions. The mice TXNIP RNA interference sequence was 5’-GGACGTGATTCCTGAAGAT-3’. A scramble form was used as a control. 5’- ATCTTCAGGAATCACGTCCAT-3’. The same sequence was employed in the *in vitro* study. One hundred microliters of each AAV9 solution (2×10^11^ vector genomes) in phosphate-buffered saline (PBS) was loaded into a 1 mL syringe attached to a 29G needle. The AAV9 solution was injected into the tail vein as previously described [21].

### Western blotting

Ten micrograms of protein were extracted from BMDMs and separated by 12% SDS-PAGE at 80 V for 1.5 h before transferring to a PVDF membrane (IPVH00010, Millipore, USA) at 300 mA for 1 h. After blocking, the membranes were incubated with primary antibodies at |4° C overnight followed by secondary antibodies at room temperature for 1 h. The primary antibodies used in the study included rabbit anti-TXNIP (1:1000, ab188865, Abcam, USA), rabbit anti-ASC (1:1000, 67824, CST, USA), rabbit anti-Cleaved Caspase-1 (1:1000, 89332, CST, USA), and rabbit anti-Caspase-1 (1:1000, 24232, CST, USA). Immunoreactive proteins were visualized using the ECL substrate kit (ab65623, Abcam, USA). ACTB was used as an internal control.

### Statistical analysis

Data analysis was performed using SPSS 19.0 software. The Shapiro-Wilk test assessed whether data conformed to a normal distribution. Statistical analysis was performed using the Pearson Chi square test (n ≥ 5) or Fisher exact test (n < 5) with subsequent multiple comparisons using Chi square with Bonferroni correction for categorical variables. One-way ANOVA with subsequent post-hoc multiple comparisons using the Student-Newman-Keuls test for continuous variables was also used for analysis. The Kruskal–Wallis test was applied to nonparametric testing of multiple independent samples with a Dunn-Bonferroni test for post-hoc comparisons.

### Ethics approval and consent to participate

The clinical study was approved by the hospital ethics review board of Shanghai Ninth People’s Hospital, Shanghai Jiao Tong University School of Medicine. All animal experiment procedures after appraisals and feasibility studies were approved by the Shanghai Ninth People’s Hospital institutional ethics committee (SH9H-2018-A45-2). All experimental contents were in accordance with the guidelines of the Directive 2010/63/EU of European Parliament.

### Availability of data and material

The data that support the findings of this study are available from the corresponding author upon reasonable request.

## Supplementary Material

Supplementary Figure 1

Supplementary Table 1
