# Supplementary material for: Nicotine exacerbates atherosclerosis through a macrophage-mediated endothelial injury pathway
Source: Aging (Albany NY). 2021 Feb 24;13(5):7627–43. doi: 10.18632/aging.202660 (PMC7993665; doi:10.18632/aging.202660)
Supplement: Supplementary Figure 1 [file aging-13-202660-s001.pdf]

SUPPLEMENTARY FIGURE

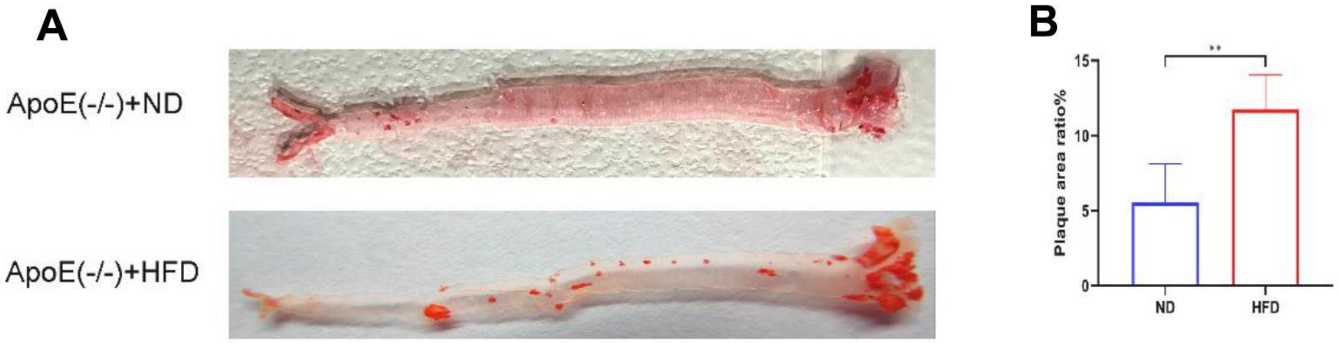

Supplementary Figure 1. (**A**, **B**) Atherosclerotic lesion areas in aorta of ApoE-/- mice fed with HFD and normal diet. ApoE -/- mice were administrated with HFD mentioned in manuscript and normal diet (ND,4% fat) for 12 weeks. Oil Red O staining in longitudinal-section of aorta of ApoE -/- mice was carried out. The result demonstrated that atherosclerotic lesion areas in aorta of ApoE-/- mice fed with HFD was larger than that of ApoE-/- mice fed with ND(P=0.004).
