# Supplementary material for: Nicotine exacerbates atherosclerosis through a macrophage-mediated endothelial injury pathway
Source: Aging (Albany NY). 2021 Feb 24;13(5):7627–43. doi: 10.18632/aging.202660 (PMC7993665; doi:10.18632/aging.202660)
Supplement: Supplementary Table 1 [file aging-13-202660-s002.pdf]

SUPPLEMENTARY TABLE

Supplementary Table 1. Primer sequences.

TXNIP(Human):	
Forward Primer	TGTGTGAAGTTACTCGTGTCAAA
Reverse Primer	GCAGGTACTCCGAAGTCTGT
GAPDH(Human):	
Forward Primer	TGTGGGCATCAATGGATTTGG
Reverse Primer	ACACCATGTATTCCGGGTCAAT
